# Curcumin Supplementation Improves Growth Performance and Anticoccidial Index by Improving the Antioxidant Capacity, Inhibiting Inflammatory Responses, and Maintaining Intestinal Barrier Function in *Eimeria tenella*-Infected Broilers

**DOI:** 10.3390/ani14081223

**Published:** 2024-04-18

**Authors:** Yan Chen, Liheng Liu, Longfei Yu, Shuo Li, Nianhua Zhu, Jinming You

**Affiliations:** 1 Jiangxi Province Key Laboratory of Animal Nutrition, College of Animal Science and Technology, Jiangxi Agricultural University, Nanchang 330045, China; chenyan196@sina.com (Y.C.); ylf949525707@sina.com (L.Y.); lishuo199x@163.com (S.L.); 2 College of Animal Science and Technology, Jiangxi Agricultural University, Nanchang 330045, China; lihengliu0714@163.com

**Keywords:** anticoccidial index, broilers, curcumin, *Eimeria tenella*, growth performance, intestine

## Abstract

**Simple Summary:**

Information about the effect of curcumin supplementation on the cecum of *Eimeria tenella*-infected broilers is scarce. This study examined the effects of curcumin on the growth performance, antioxidant system, intestinal integrity, and microbiota structure of broilers infected with *E. tenella*. Thus, this study provided a reference for using curcumin as a healthy feed additive.

**Abstract:**

This study was conducted to investigate the effects of dietary curcumin supplementation on growth performance, anticoccidial index, antioxidant capacity, intestinal inflammation, and cecum microbiota in broilers infected with *Eimeria tenella*. A total of 234 one-day-old broilers were categorized into three treatments, with six replicates per treatment containing 13 broilers each. The three treatments included the control group, *Eimeria tenella* group, and *Eimeria tenella* + curcumin (200 mg/kg) group. The feeding trial lasted for 42 days, during which the broilers were orally administered with 0.9% saline or 5 × 10^4^ *Eimeria tenella* oocysts on day 14 of the study. On day 17 and day 21, one bird per replicate was selected for slaughtering. Results indicated an increased survival rate and anticoccidial index and improved productive performance in coccidia-infected broilers with curcumin supplementation. Furthermore, curcumin enhanced the serum antioxidant capacity in *Eimeria tenella*-infected broilers, evidenced by increased serum catalase activity (3d, 7d), as well as decreased malondialdehyde level (3d, 7d) and nitric oxide synthase activity (7d) (*p* < 0.05). Curcumin also improved intestinal inflammation and barrier function, evidenced by the downregulation of *interleukin (IL)-1β* (3d, 7d), TNF-alpha *(TNF-α)* (3d, 7d), and *IL-2* (7d) and the up-regulated mRNA levels of *claudin-1* (7d), *zonula occludens* (*ZO-1*; 3d, 7d), and *occludin* (3d, 7d) in the ceca of infected broilers (*p* < 0.05). *Eimeria tenella* infection significantly disrupted cecum microbial balance, but curcumin did not alleviate cecum microbial disorder in broilers infected with *Eimeria tenella*. Collectively, curcumin supplementation enhanced growth performance and anticoccidial index in *Eimeria tenella*-infected broilers via improving antioxidant ability and cecum inflammation without affecting cecum microbiota.

## 1. Introducti0on

Poultry coccidiosis, the most serious illness affecting poultry globally, is brought on by the apicomplexan parasite *Eimeria* and is characterized by enteritis [1]. Poultry coccidiosis leads to substantial economic losses worldwide because of its high pathogenicity and transmission capability [2]. *Eimeria tenella* (*E. tenella*) is the most pathogenic among the common *Eimeria* species infecting chickens [3]. The invasion of *E. tenella* destroys the intestinal mucosal barrier and causes cecum swelling and bleeding and the excretion of large amounts of bloody stool after infection [4,5]. Intestinal damage due to coccidiosis can also lead to increased intestinal permeability, reduced digestibility, poor growth rate, and, subsequently, the death of severely infected chickens [6,7]. Currently, the main drugs used to control Eimeria infection include polyether ionophores, antibiotics, and chemically synthesized drugs [8]. However, the excessive use of antibiotics causes bacterial resistance and drug residue issues. Therefore, developing efficient and economical treatments for diseases caused by *E. tenella* remains an urgent need.

In recent years, plant extracts have been considered as potential anticoccidial agents and have attracted the attention of researchers. Several studies have highlighted plant extracts’ [9] immunoregulatory, antioxidant, and anti-inflammatory activities [10,11]. Curcumin, a bioactive compound from turmeric rhizomes, possesses significant anti-inflammatory and antioxidant properties, regulating the body’s immune functions [12,13]. Zhang et al. (2018) reported that curcumin can increase the glutathione peroxidase content in poultry to clear reactive oxygen species (ROS) generated by oxidative stress [14]. Alizadeh et al. (2019) demonstrated the efficacy of curcumin as a free radical scavenger and inhibitor of malondialdehyde (MDA) production [15]. Curcumin also could improve gut morphology, maintain gut microbiome balance, and interact with the host to enhance immunity [16]. Moreover, curcumin also has antiparasitic capabilities. Curcumin can destroy sporozoites of Eimeria and reduce oocyst shedding and subsequent intestinal damage [17,18]. Additionally, the in vivo efficacy of curcumin against coccidiosis has been demonstrated in rabbits and sheep [19,20].

Various experimental models of inflammation have demonstrated the beneficial health effects of curcumin, and studies have also demonstrated curcumin’s anticoccidial effects [12,21]. However, the effect of curcumin on the main intestinal segment (cecum) of *E. tenella* has not been explored. Therefore, the cecum was chosen as the target of this study. This study examined the effects of curcumin on the growth performance, antioxidant system, intestinal integrity, and microbiota structure of broilers infected with *E. tenella*. Thus, this study provided a reference for using curcumin as a healthy feed additive.

## 2. Materials and Methods

### 2.1. Oocyst Procurement

The spore oocysts of *E. tenella* were donated by the Animal Pharmacy Laboratory of Jiangxi Agricultural University. Thirty broiler chickens (14 days old) free of coccidiosis were gavaged with 5 × 10^4^ of sporulated *E. tenella* oocysts to propagate the oocysts. Feces from infected chicks were collected 7 days post-infection for oocyst isolation. The oocysts were sporulated in 2.5% (*w*/*v*) potassium dichromate solution at 28 °C for 3 days and then washed thrice with physiological saline [22,23].

### 2.2. Birds and Their Management

Curcumin, a 95% natural turmeric extract, was procured from Sensei Herbal Essence Company for this study. The curcumin dose in this study was based on the poultry industry’s recommended dose of 200 mg/kg [21]. Curcumin powder was weighed and added to the basal diet, and then the diet was mixed thoroughly. A total of 234 one-day-old males of a locally bred hybrid strain of broilers (Jiangxi Province) were selected. They were randomly categorized into three groups, with six replicates per group, each containing 13 broilers. The experimental groups were as follows: control group (CT), broilers were fed basal diet; coccidiosis group (CTE), broilers were fed the basal diet and were infected with 5 × 10^4^ *E. tenella* oocysts when they were 14 d old; curcumin + coccidium group (CUE), broilers were fed the basal diet with 200 mg/kg curcumin and were infected with 5 × 10^4^ *E. tenella* oocysts when they were 14 days old. Broilers in the control group received an equivalent volume of phosphate-buffered saline via gavage. One replicate was randomly selected from each of the three treatment groups to be repeated as the anticoccidial index (ACI) group. Each chicken in the ACI group was weighed and labeled on day 14, and they were weighed again on day 21 to calculate the 7 day weight gain. At the same time, the number of chick deaths from 1 to 7 days after infection with coccidia was recorded, and the mortality rate during this period was calculated. The remaining 5 replicate groups were used to calculate production performance. The average daily gain (ADG), average feed intake (ADFI), and the ratio of feed to meat (FRC) were calculated when the broilers were 14, 21, and 42 days old.

Chickens had ad libitum access to a consistent corn–soybean basal diet meeting NRC (1994) nutrient recommendations for 42 days. The dietary composition and nutrient level are summarized in Table 1. The feeding environment was strictly disinfected and free of any coccidial contamination.

### 2.3. Sample Collection

On day 17 (3 days post-inoculation–dpi) and day 21 (7 dpi), except for the ACI group, one bird per cage was randomly selected from each replicate. Before sampling, the live weights of the chicks were measured, and blood was collected from the left brachial vein. Then, cervical dislocation was performed to euthanize the broilers. The middle part of the cecum (2 cm) was taken and fixed in a paraformaldehyde solution. After slicing, hematoxylin and eosin (H–E) staining was used to observe the intestinal morphology. Cecal contents were collected and stored at −80 °C after snap-freezing in liquid nitrogen. The blood was centrifuged for 15 min (3000 RPM, 4 °C), and the supernatant was extracted and kept at −20 °C.

### 2.4. Estimation of the Anticoccidial Index

The anticoccidial index (ACI) was calculated as follows: (relative weight gain rate + survival rate) − (oocysts index in cecum + gross lesions score). On day 21 (7 dpi), each chicken in the ACI group of the three treatment groups was weighed, and daily gain for the 7 days infected with coccidia was calculated. Cecal lesions were dissected and recorded after euthanizing all broilers by cervical dislocation, and cecal contents were collected to count coccidium oocysts. The relative weight gain rate (×100%) was the ratio of average weight gain between the infected group and the control group 0–7 dpi. The survival rate (×100%) was the ratio of the number of surviving chicks at 7 dpi in a treatment group to the initial total number of chicks. Oocyst count in feces was determined using the McMaster technique [23]. As described by Xu Song et al. (2020) [22], a lesion score was estimated according to the degree of cecal lesion.

### 2.5. Determination of Antioxidant Indicators

Serum markers were calculated in accordance with the manufacturer’s protocols (Nanjing Jiancheng Institute of Biological Engineering, China). The antioxidant indexes included total superoxide dismutase (SOD) activity, MDA, total antioxidant (T-AOC) energy, catalase (CAT), nitric oxide synthase (NOS), and glutathione peroxidase (GSH-Px).

### 2.6. Gene Expression Analyses

Total RNA was extracted from the cecal mucosa of broilers using the TransZol Up Plus RNA Kit following the manufacturer’s instructions. The purity and concentration of the isolated RNA from samples were analyzed using Nanodrop ND-1000. The quality assessment and reverse transcription of RNA samples into cDNA were performed using the EasyScript^®^One-Step gDNA Removal and cDNA Synthesis Supermix kit. The following target genes were measured in this study: *interleukin (IL)-1β*, *IL-2*, *IL-17*, tumor necrosis factor *(TNF)-α*, *occludin*, *claudin-1*, and *zonula occludens (ZO)-1*. Table 2 lists the primer sequences of the target genes. The 2^−ΔΔCT^ method was used to quantify gene expression, and *Gapdh* was used as the housekeeping gene.

### 2.7. Pyrosequencing of 16S rDNA Amplicon

The total bacterial DNA was extracted from samples using the Fast DNA SPIN extraction kit (MP Biomedicals, Santa Ana, CA, USA). The concentration and quality of the extracted DNA were estimated using a NanoDrop ND-1000 spectrophotometer and by agar gel electrophoresis. Then, the polymerase chain reaction (PCR) amplification of specific gene regions was conducted according to the method described by Jinfeng Song [24]. The amplified product was purified by Agcourt AM Pure Beads and quantified using the PicoGreen dsDNA Assay Kit. After quantification, the same amount of amplicon was collected, and the 2 × 300 bp back end was sequenced using the Illumina MiSeq platform and MiSeq Reagent Kit v3 (Shanghai Personal Biotechnology Co., Ltd., Shanghai, China).

### 2.8. Statistical Analysis

The anticoccidial index was compared between treatments using the Kruskal–Wallis nonparametric statistic, incorporating mean and percentage scores. Other data are presented as mean ± standard error of mean (SEM). Data analysis was performed using Statistical Package for the Special Sciences (SPSS) statistical software (version 25.0, Chicago, IL, USA), applying a one-way ANOVA followed by a Duncan post hoc test. The relative abundance of species was determined using the LEfSe analysis of the 10 most abundant door groups of summary data (http://huttenhower.sph.harvard.edu/galaxy).

## 3. Results

### 3.1. Curcumin Supplement Improved Growth Performance in Coccidia-Infected Broilers

This study assessed the growth performance of broilers. Table 3 displays the production performance data. From ages 1 to 14 d, there was no difference in the ADG, ADFI, and FCR among the treatments (*p* > 0.05). From ages 15 to 21 d, compared with the CT groups, birds in CTE groups exhibited significantly lower ADG and ADFI (*p* < 0.05). Compared with the CTE group, curcumin supplementation significantly improved the ADG and ADFI of broilers (*p* < 0.05). From ages 22 to 42 d, relative to the CT group, birds in CTE groups had significantly lower ADG and ADFI but higher FCR (*p* < 0.05), and relative to the CTE groups, curcumin supplementation significantly improved the ADG of broilers (*p* < 0.05) and also exhibited a tendency toward higher ADFI and lower FCR (*p* < 0.1). From ages 1 to 42 d, broilers in the CTE group had lower ADG and ADFI and higher FCR compared to the CT group (*p* < 0.05), while curcumin supplementation significantly increased ADFI and ADG and decreased FCR relative to the CTE group (*p* < 0.05).

### 3.2. Curcumin Supplementation Can Enhance the Anticoccidial Index

**Figure 1:**
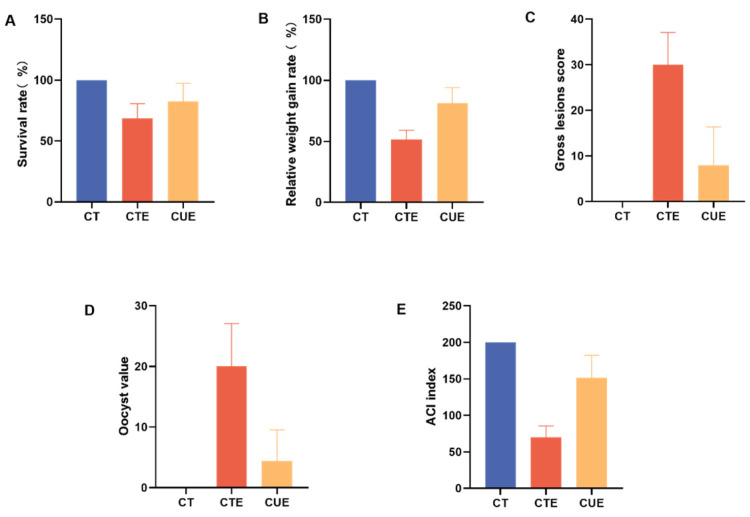
Effects of curcumin on the anticoccidial index (ACI) of broilers infected with *Eimeria tenella*. The ACI index results. (**A**) Survival rate (%); (**B**) relative weight gain rate (%); (**C**) gross lesions score (%); (**D**) oocyst value; (**E**) ACI index.

The anticoccidial effect of curcumin was evaluated by calculating the anticoccidial index. See Attachment 1 for pictures of blood stool and the cecum of some coccidia-infected broilers. The ACI index results are shown in Figure 1. Survival rates were 100% in the CT group, 68.48% in the CTE group, and 82.44% in the CUE group (Figure 1A). The relative weight gain rates are shown in Figure 1B. Compared with the CT group, the CTE group gained 51.53%, and the CUE group gained 81.31%. The CT birds had a lesion score of 0 (100%) in the gut tested for lesions; the CTE group scored 30, and the CUE group scored 8 (Figure 1C). The oocyst value was 0 in the CT group, 20 in the CTE group, and 4.4 in the CUE group (Figure 1D). According to the results, the ACI index was 200% for the CT group, 69.99% for the CTE group, and 151.34% for the CUE group (Figure 1E).

### 3.3. Curcumin Supplement Can Improve Antioxidant Capacity

**Figure 2:**
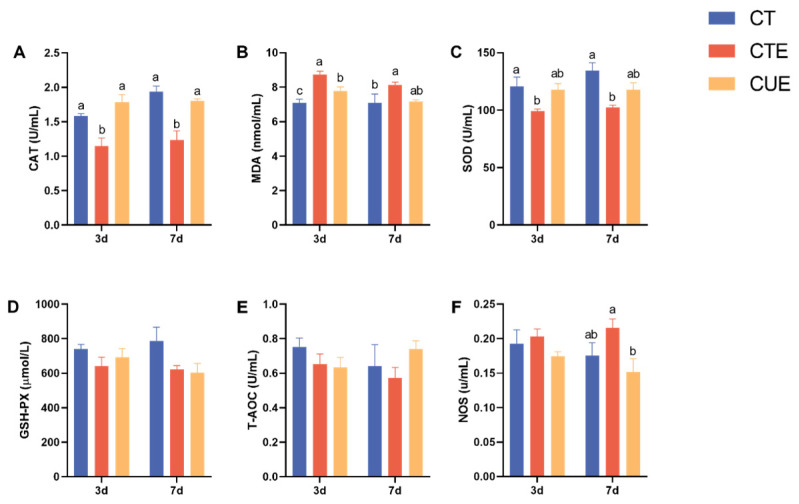
Effects of curcumin on serum antioxidant capacity parameters of broilers infected with *Eimeria tenella*. (**A**) the activity of CAT; (**B**) the concentrations of MDA; (**C**) the activity of SOD; (**D**) the activity of GSH-Px; (**E**) The degrees of T-AOC: total antioxidant capacity; (**F**) the activity of NOS. ^a–c^ Different letters indicate significant differences between respective means (*p* < 0.05).

Compared to the CT group, the CTE group demonstrated a higher MDA concentration (*p* < 0.05, at 3 and 7 dpi) and NOS activity (*p* < 0.05, at 7 dpi), but CAT and SOD activities decreased (*p* < 0.05, at 3 and 7 dpi) (Figure 2). Specifically, compared to the CTE group, curcumin supplementation significantly increased the activities of CAT (*p* < 0.05, at 3 and 7 dpi) and SOD (*p* < 0.05, at 3 dpi and 7 dpi) and decreased MDA concentration (*p* < 0.05, at 3 dpi) and NOS activity (*p* < 0.05, at 7 dpi).

### 3.4. Effect of Curcumin on the Gene Expression of Cecal Mucosal Cytokines

**Figure 3:**
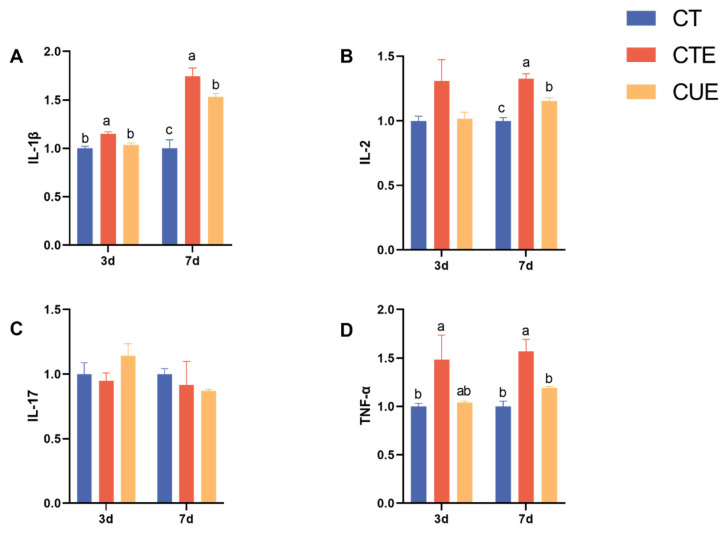
Effects of curcumin on the gene expression of cytokines in the cecal mucosa of broilers infected with *Eimeria tenella*. (**A**) the relative mRNA expression levels of *IL-1β*; (**B**) the relative mRNA expression levels of *IL-2*; (**C**) the relative mRNA expression levels of *IL-17*; (**D**) the relative mRNA expression levels of *TNF-α*. ^a–c^ Different letters indicate significant differences between respective means (*p* < 0.05).

Investigating the mechanism by which curcumin regulates the anticoccidial index involved measuring the expression of genes associated with cecal inflammatory factors. Figure 3 displays the cytokine gene expression findings in the chicken cecal mucosa. Compared with the birds in the CT group, the *Eimeria* challenge significantly promoted the mRNA levels of *TNF-α*, *IL-1β* (*p* < 0.05, at 3 and 7 dpi), and *IL-2* (*p* < 0.05, at 7 dpi) in the CTE group. Compared to the CTE group, curcumin supplementation significantly suppressed the mRNA levels of *IL-1β*, *TNF-α* (*p* < 0.05, at 3 and 7 dpi), and *IL-2* in the CUE group (*p* < 0.05, at 7 dpi). Therefore, curcumin can alleviate the inflammatory response of broilers infected with *E. tenella*.

### 3.5. Curcumin Supplementation Maintained the Integrity of the Intestinal Epithelial Barrier

**Figure 4:**
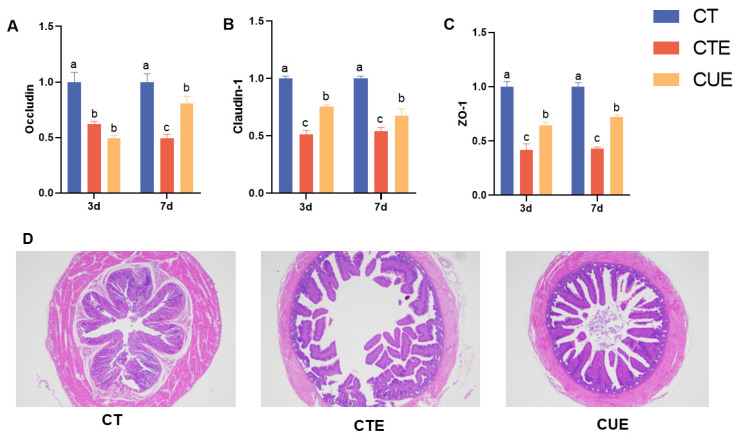
Effects of curcumin on intestinal integrity of broilers infected with *Eimeria tenella*. (**A**) the relative mRNA expression levels of *occludin*; (**B**) the relative mRNA expression levels of *claudin-1*; (**C**) the relative mRNA expression levels of *ZO-1*; (**D**) sections of cecal tissues. ^a–c^ Different letters indicate significant differences between respective means (*p* < 0.05).

This study examined curcumin’s impact on the gene expression of tight junction proteins and the morphological structure of the cecum in broilers infected with coccidia, assessing the integrity of intestinal barrier function. Compared with the birds in the CT group, the mRNA levels of *claudin-1*, *occludin*, and *ZO-1* were suppressed in the challenged birds (*p* < 0.05, at 3 and 7 dpi) in the CTE group (Figure 4). Compared to the CTE group, curcumin supplementation significantly enhanced the mRNA levels of ZO-1 (*p* < 0.05, at 3 and 7 dpi) and occludin (*p* < 0.05, At 7 dpi) in the CUE group. H–E-stained cecum sections are presented in Figure 4D. In the CT group, a clear cecum tissue structure was observed, with the villi arranged in an orderly manner, normal cell morphology, and a clear boundary. The cecal tissue structure was severely damaged, the villi were broken, more inflammatory cells had infiltrated, and the Eimeria oocysts were more tender in the CTE group. The tissue structure of the CUE group was normal, the villi were complete, and the cecal lamina propria was clear. These results suggest that curcumin supplementation maintained the integrity of the intestinal epithelial barrier.

### 3.6. Effect of Curcumin on Cecal Microbiota Structure

**Figure 5:**
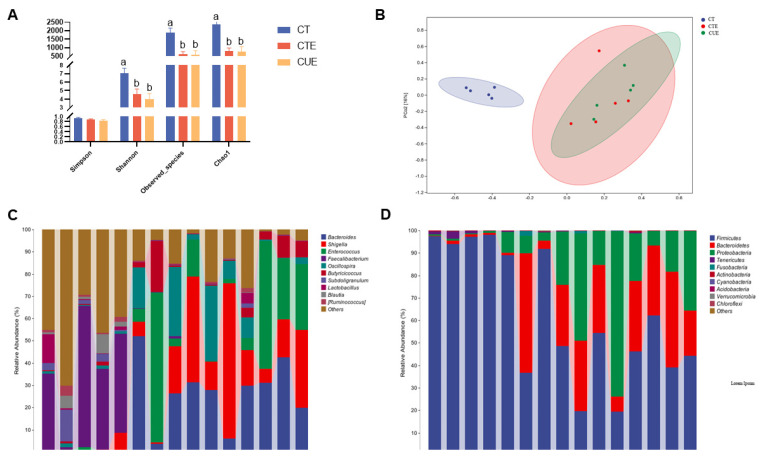
Effect of curcumin on cecal microbiota of broilers infected with *Eimeria tenella*. (**A**) The *diversity index* of cecal flora. (**B**) Principal coordinate analysis. (**C**) Cecal microbiota composition at phylum levels in broilers infected with *Eimeria tenella*. (**D**) Cecal microbiota composition at genus levels of broilers infected with *Eimeria tenella*. ^a,b^ Different letters indicate significant differences between respective means (*p* < 0.05).

Here, we attempted to explore whether supplementation with curcumin may play a key role in cecal microbiota structure. The microbial richness and diversity in the cecum microbiota on day 21 are presented in Figure 5. Relative to the CT group, the CTE and CUE groups showed significant reductions in the Chao1 index, observed species, and Shannon indices in the cecum microbiota on day 21 (*p* < 0.05) (Figure 5A). The alpha indices did not differ significantly between the CTE group and CUE group. The principal coordinate analysis analyses based on taxa summary data showed distinct clusters of the microbiota community from the ceca of the CT group, the CTE group, and the CUE group (Figure 5B).

The relative abundance of bacterial species in the gut microbiota is presented in Figure 5C and Table 4. A total of 10 phyla were detected in the ceca of broilers in the CT group. These included Firmicutes, Proteobacteria, Bacteroidetes, Tenericutes, Fusobacteria, Actinobacteria, Cyanobacteria, Acidobacteria, Verrucomicrobia, and Chloroflexi. According to sequencing data, Firmicutes accounted for 95.10% of all operational taxonomic units (OTUs), followed by Proteobacteria, Bacteroidetes, and Tenericutes. Compared to the CT group, the relative abundance of Firmicutes, Proteobacteria, and Tenericutes decreased from 95.09% to 50.26% and 42.25%, while that of Proteobacteria and Bacteroidetes increased (from 1.02% to 29.10% and 26.34% and from 2.34% to 19.60% and 31.01%, respectively) in the cecal microbiota of chicken in the CTE and CUE groups. At the genus level, Faecalibacterium was the dominant taxon (36% of OTUs) in the CT group, while Lactobacillus accounted for 3%, Ruminococcus 1.8%, Shigella 1.6%, and Oscillospira 1.2%. Compared with the CT group, the CTE and CUE groups exhibited increased relative abundances of Bacteroides and Oscillospira, and that of Enterococcus and Blautia decreased in the cecum of the chickens. The cecal flora abundance of the CTE and CUE groups did not differ significantly; only the relative abundance of *Oscillospira* decreased in the CUE group. This study showed that dietary curcumin supplementation reduced the relative proportion only of *Oscillospira* caused by coccidia infection.

## 4. Discussion

Coccidia infection leads to significant pathological damage in broilers, notably causing extensive cecal bleeding and mortality. In the current study, chickens infected with *E. tenella* exhibited swelling and bleeding, along with many egg sacs in the cecum. After the 3 dpi of coccidium oocysts infection, the chickens began to display slight hematochezia symptoms and decreased feed intake. After 5 dpi, chicks in the infected group began to die. The survival rate of the coccidia group was 68.48%, indicating that the coccidium oocysts were highly toxic, subsequently causing serious infection in all chicks, which was consistent with that reported in many previous studies [4,5]. This experiment recorded an ACI value of 151.34% for the CUE group, indicating a significant anticoccidial effect, in contrast with 35.44% for the CT group. Concurrently, chickens fed a curcumin-supplemented diet had lower lesion scores and decreased fecal oocyst output at 7 dpi. Kim et al. (2013) also reported that curcumin could enhance resistance to coccidiosis, promote weight gain, and reduce oocyst loss and intestinal damage [25]. Curcumin can destroy *E. tenella* sporozoites, thereby reducing their colonization in the gut [17]. These results suggest that curcumin supplementation can improve the survival rate of coccidia-infected chickens and alleviate adverse symptoms. In summary, *E. tenella* infection can seriously damage chicks, while adding curcumin to feed can improve the survival rate of these chickens, alleviate the adverse symptoms caused by the infection, and maintain their health.

Organismal damage can impair nutrient absorption, affecting the animal’s performance. The current study found that *E. tenella* significantly reduced ADG and ADFI in chickens during the infectious stage, aligning with the findings by Rochell et al. (2017) [26], with 9% and 4% reductions in ADG and ADFI, respectively, in coccidia-infected chickens. Similarly, Teng et al. (2020) reported that the coccidiosis infection of broilers significantly reduced their growth performance, metabolizable energy, and intestinal morphology [27]. The worsened growth performance during coccidiosis may be attributed to reduced intestinal integrity, which leads to the impaired digestion and absorption of nutrients from the intestine, a common phenomenon in hosts with parasitic infestation. Previous studies have reported that the curcumin supplementation of broiler diets improved growth performance [28]. In this study, curcumin supplementation alleviated the negative effects of coccidiosis on performance parameters. Subsequently, it improved daily weight gain and the feed intake of broilers aged 15 to 21 days and 1 to 42 days. This finding was consistent with Yadav et al. (2020) [21]. This could be due to a larger villus area induced by curcumin feeding for longer, improving nutrient absorption in the later phase [28]. It has been found that curcumin supplementation can significantly increase villus height-to-crypt-depth ratio (VCR) and improve the morphology of ileal mucosa to repair intestinal damage in piglets [29]. It has also been reported that curcumin improved nutrient metabolism by enhancing the production of bile acids and the activity of gastric enzymes to accelerate digestion and absorption [30]. In conclusion, under the coccidium challenge, chickens fed with a curcumin-supplemented diet could alleviate the decline in growth performance.

In the present study, compared to the non-challenged birds, those that received the challenge exhibited a higher MDA concentration, NOS activity, and lower SOD and CAT activities. This finding suggests that coccidiosis in poultry led to an imbalance between the oxidative and antioxidant systems in the body, leading to the generation of large amounts of free radicals of oxygen and oxidative stress. This observation aligns with findings by Georgieva et al. (2010) [31], noting increased plasma MDA concentrations and decreased blood SOD activity in coccidia-infected birds. The decrease in antioxidative enzyme activity worsened cellular resistance against oxidative cell damage and led to cell death. Results indicated that curcumin supplementation significantly decreased MDA levels while enhancing CAT and SOD levels in the serum of coccidia-infected broilers, aligning with the findings by Zhang et al. (2018) [14]. Curcumin has often been reported to exhibit a direct antioxidant capacity, which can relieve oxidative stress by scavenging ROS [32,33]. These could be attributed to the chemical structure of curcumin; a lipophilic polyphenol with a conjugated double bond serves as a powerful electron donor to inhibit the redox processes that produce ROS [34,35]. Thus, curcumin could improve the antioxidant capacity of broiler chickens infected with Coccidioides and maintain the equilibrium of the antioxidant enzyme system.

Coccidia colonizes the cecum and causes cecum bleeding, structural destruction, and inflammatory response [4]. *TNF-α* and IL are two important cytokines involved in the body’s immune and inflammatory responses [36]. Results indicated that coccidial infection led to significant cecal bleeding and elevated levels of pro-inflammatory factors such as *IL-1β*, *IL-2*, and *TNF-α*. Likewise, many studies have reported the enhanced expression of pro-inflammatory cytokines in response to chicks infected with coccidia [37,38,39]. The excessive production of pro-inflammatory cytokines causes damage to the body, while the decreased expression of pro-inflammatory cytokines can effectively alleviate the body’s inflammatory response [40]. In line with earlier research [41], experimental results indicated a reduction in the mRNA expression of *TNF-α* and *IL-1β* genes in the chicken cecal mucosa due to curcumin supplementation. Curcumin can inhibit the signal transduction of some pro-inflammatory enzymes and inflammation-inducing transcription factors, thus reducing the body’s inflammatory response [42,43]. Curcumin inhibits IκBα phosphorylation and degradation, thereby blocking the activation of the nuclear factor kappa B (NF-κB) transcription factor and its p65 subunit’s nuclear translocation, leading to anti-inflammatory effects [44]. Therefore, curcumin possibly inhibits the pro-inflammatory response of the body by inhibiting the expression of pro-inflammatory factors, thus alleviating the inflammatory damage caused by coccidia.

In the current study, the relative expression of tight junction protein mRNAs, including claudin-1, occludin, and ZO-1, was down-regulated in coccidia-infected chicks. These results suggest that coccidiosis infection impairs intestinal barrier function by disrupting tight junctions, consistent with Kaingu et al. (2017) [4]. According to some studies, curcumin supplementation to the diet can reduce adverse inflammatory effects on the intestinal morphology of chickens. Likewise, in this study, curcumin supplementation could increase the mRNA expression of *Occludin* and *Claudin-1* in the cecal mucosa, indicating enhanced intestinal barrier functions. This was also reported by Wang et al. (2017), who showed that curcumin can improve intestinal barrier function by regulating intracellular signal transduction and tight junction connectivity [45]. Our analysis of pathological lesions further confirmed the protective effect of curcumin. Cecal tissue sections stained with hematoxylin and eosin showed that curcumin could significantly reduce the inflammatory cell infiltration caused by coccidia infection and alleviate the pathological injury of the cecum. Therefore, by promoting the expression of the tight junction protein gene, curcumin may maintain the integrity of the intestinal epithelial barrier, alleviate the intestinal damage in the cecum of broiler chickens caused by *E. tenella* infection, and lead to increased survival.

The gut microbiota plays a decisive role in the host’s health and physiology [46]. The preliminary analysis of richness and diversity indicators showed that the Chao1 index, the Shannon index, and the observed species index of the *E. tenella* infection were significantly reduced. After *E. tenella* infection in broilers, the relative abundance of *Firmicutes* significantly decreased, and that of bacteroidetes significantly increased in the cecum, which may be one of the causes of weight loss, malnutrition, and anemia in chickens [47,48,49]. In this study, curcumin had little effect on the intestinal flora structure, and the abundance of Oscillospira was reduced in the cecum of coccidia-infected broilers. Metagenomic research indicates that *Oscillospira* may break down animal-originating glycans, like glucuronic acid, requiring energy to regenerate these vital components of intestinal mucins and resulting in energy loss [50]. Therefore, a reduced abundance of *Spirochaeta oscillosum* in the cecum could enhance energy utilization efficiency in broilers. Similarly, reports of decreased *Oscillospira* abundance suggest that reducing oxidative stress supports host health maintenance [51]. This aligns with our findings, which demonstrate dietary curcumin’s potential to improve the antioxidant capacity of coccidia-infected broilers. Many studies have found that curcumin can alter the structure of the flora, increasing the abundance of potentially beneficial bacteria and reducing the abundance of pathogenic bacteria [16,52]. Collectively, *E. tenella* destroys the structure of cecal microbiota, but the cecum flora is not a key target for the curcumin treatment of coccidiosis in broiler chickens.

## 5. Conclusions

In summary, curcumin supplementation could reduce the death and the decline in the production performance of broiler chickens caused by *E. tenella* infection. This may have been achieved by enhanced antioxidant capacity, alleviating the inflammatory response, promoting the mRNA expression of tight junction proteins, and maintaining the integrity of the intestinal epithelial barrier of coccidia-infected broiler chickens.

## Figures and Tables

**Table 1:** The compositions and nutrient levels of the basal diet.

Ingredients	Content (%)	Calculated Nutrient Levels	
Corn	60.50	Metabolizable energy (MJ/kg)	12.95
Soybean meal (46% CP)	30.00	Crude protein (%)	19.17
Fish meal (67% CP)	1.80	Ca (%)	0.90
Soybean oil	3.50	Available phosphorous	0.65
Limestone	1.60	SID methionine (%)	0.40
Dicalcium phosphate	1.45	SID lysine (%)	1.00
NaCl	0.36	SID threonine (%)	0.72
DL-methionine	0.06	Methionine + Cystine (%)	0.76
L-lysine hydrochloride (98.5%)	0.03		
Mineral premix ^b^	0.50		
Vitamin premix ^a^	0.20		
Total	100		

^a^ The vitamin premix supplied the following per kilogram of complete feed: vitamins (A, 12,500 IU; D_3_, 2500 IU; K_3_, 2.65 mg; B_1_, 2 mg; B_2_, 6 mg; B_12_, 0.025 mg; E, 30 IU); biotin, 0.0325 mg; folic acid, 1.25 mg; pantothenic acid, 12 mg; and niacin, 50 mg. ^b^ The mineral premix provided the following (per kilogram of diet): manganese, 100 mg; zinc, 75 mg; iron, 80 mg; copper, 8 mg; selenium, 0.25 mg; and iodine, 0.35 mg. SID: standardized ileal digestible.

**Table 2:** Primer sequences of real-time polymerase chain reaction.

Target	Primer Sequence (5′-3′)	Accession on.	Product Size (bp)
Gapdh	F: GTGAAAGTCGGAGTCAACGG R: CGTTCTCAGCCTTGACAGTG	NM_001289745.3	184
IL-1β	F: GCATCAAGGGCTACAAGCTC R: GTCCAGGCGGTAGAAGATGA	XM_015297469.1	134
IL-2	F: TCGAGCTCTACACACCAACT R: CTTGCATTCACTTCCGGTGT	M_015276098.2	197
IL-17	F: GAGCCAGAGAGCCTCTTCAA R: TGTGGTCCTCATCGATCCTG	NM_204460.1	181
TNF-alpha	F: CTGATGGCGTGAAGAAGGTC R: GAAGAGTTCATTCGCGGCTT	NM_205149.1	95
Claudin-1	F: TACAGCCCTTGGCCAATACA R: CCAAGAAACAACCACCAGCA	NM_001013611.2	171
Occludin	F: CCTCATCGTCATCCTGCTCT R: GGTCCCAGTAGATGTTGGCT	XM_025144248.1	95
ZO-1	F: GAGCTCACAAGCTACGCAAA R: ACTTGTAGCACCATCTGCCA	XM_015278981.2	161

F: upstream primer; R: downstream primer.

**Table 3:** Effects of curcumin on the growth performance of broilers infected with *Eimeria tenella* ^1,2^.

	Items	CT	CTE	CUE	SEM	*p*-Value
1–14 d	ADG (g/d)	14.04	14.23	13.89	1.67	0.736
	ADFI (g/d)	27.60	27.02	26.06	0.36	0.219
	FCR	1.96	1.90	1.88	0.02	0.385
15–21 d	ADG (g/d)	16.92 ^a^	10.72 ^c^	15.23 ^b^	0.86	0.001
	ADFI (g/d)	34.99 ^a^	28.74 ^b^	35.00 ^a^	1.01	0.004
	FCR	2.12 ^b^	2.72 ^a^	2.30 ^ab^	1.06	0.044
22–42 d	ADG (g/d)	30.75 ^a^	26.10 ^c^	28.43 ^b^	0.63	0.002
	ADFI (g/d)	75.60	69.80	74.50	1.08	0.051
	FCR	2.46	2.68	2.62	0.42	0.073
1–42 d	ADG (g/d)	20.57 ^a^	17.01 ^c^	19.18 ^b^	0.44	<0.000
	ADFI (g/d)	46.06 ^a^	41.85 ^b^	45.18 ^a^	0.57	0.001
	FCR	2.24 ^c^	2.46 ^a^	2.35 ^b^	0.02	0.03

^1^ ADG = average daily gain; ADFI = average daily feed intake; FCR = feed conversion ratio. ^2^ SEM: standard error of the mean. ^a–c^ Different letters within a row indicate significant differences between respective means (*p* < 0.05).

**Table 4:** Cecum bacteria composition (phylum and genus level) of broilers.

Items	CT	CTE	CUE	SEM	*p*-Value
Phylum level					
Firmicutes	95.095 ^a^	50.259 ^b^	42.251 ^b^	7.554	0.001
Proteobacteria	1.021 ^b^	29.105 ^a^	26.342 ^a^	4.557	0.008
Bacteroidetes	2.337 ^b^	19.599 ^ab^	31.009 ^a^	5.370	0.080
Tenericutes	1.234 ^a^	0.011 ^b^	0.062 ^b^	0.226	0.029
Fusobacteria	0.008	0.666	0.024	0.147	0.108
Actinobacteria	0.197	0.220	0.180	0.035	0.909
Cyanobacteria	0.018	0.025	0.013	0.004	0.535
Acidobacteria	0.020	0.012	0.011	0.003	0.487
Verrucomicrobia	0.014	0.005	0.007	0.003	0.476
Chloroflexi	0.009	0.009	0.002	0.002	0.279
Others	0.047	0.090	0.100	0.023	0.634
Genus level					
Bacteroides	0.007 ^b^	0.282 ^a^	0.258 ^a^	0.045	0.009
Shigella	0.016 ^b^	0.177 ^ab^	0.288 ^a^	0.052	0.096
Faecalibacterium	0.003	0.188	0.243	0.056	0.199
Enterococcus	0.360 ^a^	0.003 ^b^	0.001 ^b^	0.055	0.001
Oscillospira	0.012 ^b^	0.172 ^a^	0.042 ^ab^	0.029	0.045
Butyricicoccus	0.007	0.052	0.051	0.016	0.437
Subdoligranulum	0.047	0.001	0.005	0.009	0.085
Lactobacillus	0.031	0.002	0.010	0.009	0.396
Blautia	0.037 ^a^	0.000 ^b^	0.001 ^b^	0.006	0.015
Ruminococcus	0.018	0.005	0.005	0.003	0.100
Others	0.461 ^a^	0.118 ^b^	0.095 ^b^	0.053	0.001

^a,b^ Different letters within a row indicate significant differences between respective means (*p* < 0.05).

## Data Availability

The data that support the conclusion of this study will be available from the corresponding author upon reasonable request.
